# Plant Responses to Brief Touching: A Mechanism for Early Neighbour Detection?

**DOI:** 10.1371/journal.pone.0165742

**Published:** 2016-11-09

**Authors:** Dimitrije Markovic, Neda Nikolic, Robert Glinwood, Gulaim Seisenbaeva, Velemir Ninkovic

**Affiliations:** 1 Department of Crop Production Ecology, Swedish University of Agricultural Sciences, Uppsala, Sweden; 2 University of Banja Luka, Faculty of Agriculture, Banja Luka, Bosnia and Herzegovina; 3 Department of Chemistry and Biotechnology, Swedish University of Agricultural Sciences, Uppsala, Sweden; Henan Agricultural University, CHINA

## Abstract

In natural habitats plants can be exposed to brief and light contact with neighbouring plants. This mechanical stimulus may represent a cue that induces responses to nearby plants. However, little is known about the effect of touching on plant growth and interaction with insect herbivores. To simulate contact between plants, a soft brush was used to apply light and brief mechanical stimuli to terminal leaves of potato *Solanum tuberosum* L. The number of non-glandular trichomes on the leaf surface was counted on images made by light microscope while glandular trichomes and pavement cells were counted on images made under scanning electronic microscope. Volatile compounds were identified and quantified using coupled gas chromatography–mass spectrometry (GC-MS). Treated plants changed their pattern of biomass distribution; they had lower stem mass fraction and higher branch and leaf mass fraction than untouched plants. Size, weight and number of tubers were not significantly affected. Touching did not cause trichome damage nor change their total number on touched terminal leaves. However, on primary leaves the number of glandular trichomes and pavement cells was significantly increased. Touching altered the volatile emission of treated plants; they released higher quantities of the sesquiterpenes (E)-β-caryophyllene, germacrene D-4-ol and (E)-nerolidol, and lower quantities of the terpenes (E)-ocimene and linalool, indicating a systemic effect of the treatment. The odour of touched plants was significantly less preferred by the aphids *Macrosiphum euphorbiae* and *Myzus persicae* compared to odour of untouched plants. The results suggest that light contact may have a potential role in the detection of neighbouring plants and may affect plant-insect interactions.

## Introduction

Coexistence with neighbouring plants is one of the most important challenges faced by plants. The impossibility of escape from unfavourable growing conditions makes plants’ survival directly dependent on their sensitivity and ability to respond to subtle cues from their surroundings. Plants detect neighbouring plants by a range of cues including light quality [1, 2], sound [3] and chemicals released by roots [4] or leaves [5]. Depending on the type of signals received from their neighbours, plants show specific responses that include changes in biomass allocation, shade avoidance and volatile emission [6–9].

Mechanical stimuli are among the many cues to which plants can respond in order to quickly adapt their growth and enhance survival in a specific environment [10, 11]. Plants respond to mechanical stimuli with range of morphological, physiological and biochemical changes. Those with specialized sensory cells such as *Mimosa pudica* and Venus fly trap *Dionaea muscipula* respond immediately [12, 13], while other plants show visible morphological modifications over longer periods of time, from days to weeks [11, 14, 15]. Common plant responses to mechanical stimulation include inhibition of internode elongation, stronger and more flexible stem and increased ratio of branch to stem diameter [12].

Agricultural crops are particularly interesting because they are exposed to many different types of mechanical stimuli that include bending [16], rubbing of the stem [17] or spraying with water [18]. Hyponastic leaf movement can also cause modest mechanical stimuli through touching of leaf tips with neighbouring plants and is perceived as the earliest signal to detect future competitors [19]. Other widespread organ movements, such as circumnutation [20] and phototropism [21] can also cause touching. Evidence that these frequently occurring phenomena are involved in plant-plant interactions in nature is still lacking. However, it is possible that mechanical stimuli are a route through which plants can gain information about their neighbours.

Plants are the basis of food webs, and the effects of plant responses to their environment can impact organisms at higher trophic levels. For example, herbivorous arthropods can be highly sensitive to changes in host plant status [22], and plant responses to environmental stimuli can alter plant-arthropod interactions (e.g. [23]). These effects can contribute to natural regulation of herbivore populations and have consequences for productivity in agricultural crop systems, as has been shown for plant-plant interactions [24, 25].

The aim of the current study was to investigate whether brief mechanical stimuli affect plant morphology, physiology and interaction with insect herbivores. Using potato as a model, we tested the effect of touching on biomass allocation, glandular and non-glandular trichome occurrence, volatile chemical emissions and olfactory attraction of aphids.

## Material and Methods

### Plants and insects

As a model plant we used cultivated potato *Solanum tuberosum* L. (Solanaceae), one of the most important vegetable crops for human nutrition, cultivated in more than 100 countries [26]. Potato tubers of cultivar Sava were provided by Lantmännen, Sweden. Cut sprouting buds taken from tubers were planted singly in plastic pots (9 × 7 × 7 cm) with potting soil (Special Hasselfors garden, Hasselfors, Sweden). After 14 days, young plants were re-planted into larger 5 litre polypropylene pots (height 18 cm, diameter 23 cm) filled with the same potting soil. Distance between each pot was 70 cm. Plants were grown in a chamber maintained at 18–22°C, a light regime of L16:D8 and 70% relative humidity. Light was provided with HQIE lamps (Hortilux Schréder, HPS 400 Watt, Holland)–one lamp per square meter.

The potato aphid *Macrosiphum euphorbiae* (Thomas) and green peach aphid *Myzus persicae* (Sulzer) were grown in cultures under the same conditions as the test-plants but in different climate chambers. *Macrosiphum euphorbiae* was reared on potted potato plants *Solanum tuberosum* L. cv. King Edward while *M*. *persicae* was reared on potted rapeseed plants *Brassica napus* L. These aphid species are the most important pests of potato since they vector a number of plant -viruses [27].

### Touching treatment

Potato leaves were treated with an artificial light mechanical stimulus, starting 17 days after planting. A soft squirrel hair face brush (Rouge) (Lindex, Sweden) was used. The terminal leaves (the first leaves to come into contact with neighbours) on the top of each potato branch were gently touched from the leaf base to the top (S1 Fig). The treatment was designed to simulate contact with a neighbouring plant and isolate the mechanical component from others such as exchange of leaf chemicals. This method has been used previously to simulate natural mechanical stimuli [14, 28–30], although it cannot be assumed to fully replicate plant-plant contact under natural conditions. The treatment could also potentially simulate arthropod movement on the leaves. Treated plants were brushed in the morning, 3 hours after the start of the photoperiod, for 1 min/day.

### Plant morphological analyses

Plants used for morphological analyses were touched as described above for 17 days. The period was based on the time needed by potato plants to start to develop flowers. Potato plants were cut at ground level using scissors and then separated into stem, branches, leaves and tubers. Tubers from each plant were carefully washed of soil particles under running water. Stem, branches and leaves from each plant were separately scanned using a dual lens scanner (Epson 4490Pro). Leaf surface, stem height, total branch length and average branch diameter were calculated using WinRHIZO (Regent Instruments), an image analysis system specifically designed for plant morphological measurements. Leaves, stems and branches from each plant were separately packed into labelled aluminium bags and dried for 48 h at 70°C to constant mass dry weights. All samples spent 24 h at room temperature before they were weighed. These data were used to calculate integral morphological indices. Specific leaf area (SLA) is calculated as proportion of leaf surface to leaf dry weight. Detailed information of the morphological indices is presented in S1 Table. To describe the relationship between plant organs i.e. how the biomass fraction of one organ relates to that of the entire above ground biomass, we calculated stem mass fraction (SMF), branch mass fraction (BMF) and leaf mass fraction (LMF) (see dataset in S2 Table). The sum of leaf weight, branch weight and stem weight is presented as total above ground biomass. Root weight was not analysed due to the difficulty of separating roots from soil. The number of tubers per plant was counted, and tubers were weighed without drying.

### Determination of trichome number

Trichomes on the adaxial leaf surface were examined to determine whether mechanical stimuli affected the number of trichomes both on the touched terminal leaves and on untouched primary leaves on the same branch. Leaf samples were carefully harvested from one randomly chosen branch without causing damage to the surfaces. From the selected branch we collected two leaf discs, one from the touched terminal leaf and one from the first primary leaf (S2 Fig). The same procedure on equivalent leaves was repeated on control plants. Each treatment was represented by 10 plants. We used a hollow metal cylinder to punch out round leaf discs (surface 0.86 cm^2^) from sampled leaves. Samples were taken only on the areas between leaf veins [31].

### Light microscopy of leaf surface

Light microscopy was used to determine the number of non-glandular trichomes on the leaf surface. Leaf discs obtained as described above were placed under a light microscope (Leica MZFLIII) under magnification 1.0 × to make a digital image for further analyses. The number of trichomes per leaf disc was counted using a graphical program CorelDraw [32] in which each trichome counted on the leaf disc was carefully marked using the ‘ellipse’ tool. All ellipses were then selected using the ‘pick’ tool and the program automatically counted the number of selected objects. (see S3 Table for detailed information).

### Scanning Electronic Microscope (SEM) of leaf surface

We used SEM to determine the frequency of glandular trichomes and pavement cells on the leaf discs, and to check whether glandular trichomes were damaged by the touching treatment. One randomly chosen branch was harvested from five control and five treated plants. Leaf discs from terminal and primary leaves (obtained by hollow cylinder as described above) were attached to double-sided adhesive carbon sticker (same size as leaf discs) previously mounted on aluminium SEM stubs. Each leaf disc spent a maximum of 15 minutes inside the SEM to avoid effects of higher temperatures on the trichomes. Images made under SEM covered an area of 1 mm^2^. SEM characterization of leaf discs was carried out using a Hitachi TM-1000-μDeX environmental table top electron microscope, without coating of the samples in a charge-up reduction mode. Glandular trichomes were determined according to a classification previously described by Glass *et al*. [33] (see dataset in S3 Table).

Pavement cells were counted from SEM images where each recording was expressed as the mean cell number of three randomly chosen square fields per leaf (0.06 mm^2^ per field). The number of whole pavement cells inside each square field was estimated in CorelDraw [32] by marking each cell using the 'ellipse' tool and counting as described above. The final estimate of cell density was calculated as the mean of five independent measurements for terminal and primary leaves on treated and control plants respectively (dataset presented in S4 Table).

### Collection of volatiles

Prior to volatile collection, polyethyleneterephthalate (PET) oven bags (35 cm x 43 cm, Toppits®, Klippan, Sweden) were baked in an oven at 140°C for 2 h to remove contaminants. Glass tubes (5 mm diameter) containing Porapak Q (PPQ, 50mg of PPQ per tube, mesh 50/80, Supelco, Bellefonte, PA, USA) were rinsed with redistilled dichloromethane (DCM) and baked overnight under nitrogen flow at a temperature of 140°C and cooled to room temperature just before volatile collection started.

Plants were subjected to touching treatment as described above for 8 days and control plants were untreated. Twenty four hours after this treatment, pots containing one potato plant were carefully enclosed in PET bags, taking care not to touch the leaves and shoots. Charcoal-filtered air was pumped in at 600 ml min^-1^ and a tube containing Porapak was inserted through a hole in the top of the bag and air drawn through via PTFE tubing connected to a pump (400 ml min^-1^). The difference in flow rates created a positive pressure to minimize entry of unfiltered air. A small hole cut in the top of the bag prevented build-up of pressure. Air was pumped in for 30 minutes prior to volatile collection to flush contaminating volatiles from the system. Volatile collection was carried out for 48h under controlled environmental conditions (21°C, 16h:8h light-dark cycle). Six replicates were carried out for each treatment, and two control treatments consisting of pots and soil without plants were included.

### Chemical Analysis

Collected volatile compounds were extracted from PPQ traps with 750 μL of redistilled DCM into a 2 ml glass vial. An internal standard (1-nonene) was added to achieve a concentration of 50ng/μl in the sample. The samples were then reduced to 50 μl volume under a gentle nitrogen flow.

Compounds were identified and quantified using coupled gas chromatography–mass spectrometry (GC-MS). A 2μl aliquot of each sample was injected onto a HP-5 column (95% dimethyl polysiloxane and 5% diphenyl polysiloxane; 30 m, 0.25 mm i.d., and 0.25 μm film thickness; J&W Scientific, Santa Clara, CA, USA) housed in a 7890A gas chromatograph (Agilent Technologies, Santa Clara, CA, USA) coupled to an Agilent 5975C mass spectrometer. Ionization was by electron impact at 70 eV. The oven temperature was held at 30°C for 1min, then programmed at 10°C min^–1^ to 250°C. The carrier gas was helium with a flow rate of 1 mL min^–1^. Identifications were made by comparison of spectra with those of authentic samples in a commercial database (NIST 2008) and by comparing mass spectra and retention times with those of authentic standards where available. Only compounds appearing in the headspace of plants and not of pots with soil were quantified. Quantifications were made using the internal standard. Chemical standards were obtained as follows: (α)-pinene (98%), 6-methyl-5-hepten-2-one (99%), linalool (97%), methyl salicylate (98%), (+)-cyclosativene (99%) (all from Sigma-Aldrich, Sweden), (E)-β-caryophyllene (98.5%, Fluka, Sweden), (E)-nerolidol (>85%Fluka), hexahydrofarnesyl acetone (98%, Bedoukian, Danbury, CT, USA). Standards of (E)-ocimene, (E,E)-4,8,12-Trimethyl-1,3,7,11-tridecatetraene (TMTT) and (E)-4,8-dimethyl-1,3,7-nonatriene (DMTT) were kindly provided by Dr Mike Birkett, Rothamsted Research, UK. Standards were not available for (α)-copaene, cadinene, germacrene D-4-ol, and two unidentified compounds assumed to be sesquiterpenes based on their retention indices and mass spectra (unknown sesquiterpene 1 (m/z 161, 105, 91, 119, 177, 204); unknown sesquiterpene 2 (m/z 161, 119, 134, 105, 204)). Germacrene D and germacrene D-4-ol have been previously reported as volatiles from potato leaves [34, 35] (see dataset in S5 Table).

### Aphid olfactory response

Plants used for olfactory bioassays were exposed to touching treatment for 8 days as described above. Aphid olfactory preference was tested using a Perspex two way airflow olfactometer consisting of two stimulus zones (length 4 cm) directly opposite each other connected by a neutral central zone (2.5 × 2.5 cm) [5]. Olfactometry experiments were done 24 h after the last touching treatment. One arm of each olfactometer was connected to a cage containing a touched potato plant and the other arm to a cage containing an untouched potato plant. The position of the treatments in the two-arm olfactometer was switched between the left and right arms in each olfactometer to minimize positional bias. Airflow in the olfactometer was 250 ml/min, which established discrete air currents in the stimulus zones.

Tested aphids were randomly collected from cultures using a fine paintbrush and placed in Petri dishes with moistened filter paper to prevent dehydration. Aphids were left in the bioassay room for 1 h to acclimatize prior to the experiments. A single aphid was introduced into the central zone of the olfactometer through a hole in the top and, after an adaptation period of 10 min, the position of the aphid in the arms, defined as a visit, was recorded at 3 min intervals over a 30-min period. The accumulated number of visits of a single aphid in a single arm after ten recordings was regarded as one replicate. Data were expressed as mean of individual aphid visits per olfactometer arm during the observation period of 30 min (dataset in S6 and S7 Tables).If an aphid did not move between three consecutive observations, the replicate was discarded and these individuals were not included in the analysis. To avoid pseudoreplication individual aphids were used only once. Olfactometers were washed with 10% Teepol L (TEEPOL, Kent, UK) and rinsed with 80% ethanol solution and distilled water and left to air dry. The number of replicates (individual aphids tested) was 25.

### Statistical analyses

Differences in morphological parameters between touched and untouched plants were tested by t-test. Using histogram and plots, data values and their residuals were found to be normally distributed and homoscedastic. Values not normally distributed were log-transformed. One way ANOVA was used to determine differences between treatments in the number of pavement cells on terminal and primary leaves followed by Tukey's HSD test for pairwise comparisons. Two way ANOVA was used to assess differences between treatments in the number of trichomes on terminal leaves and primary leaves. The square root transformation was applied to normalize the data and independence of mean and variance. Pairwise comparisons of means were made using Tukey’s HSD test.

Principal component analyses (PCA) was used to reduce the complexity of multivariate volatile data and to determine if there were overall differences between the peak areas of volatile compounds released from touched and control potato plants. The first two principal components PC1 and PC2 biplots of the total PC scores and loadings were used to visualize the results. T- test was used to determine significant differences between individual volatile compounds of treated and control plants. Data from olfactory bioassays were analysed with Wilcoxon’s matched pairs tests. Analyses were performed with the Dell Statistica software [36].

## Results

### Pattern of biomass distribution

**Fig 1:**
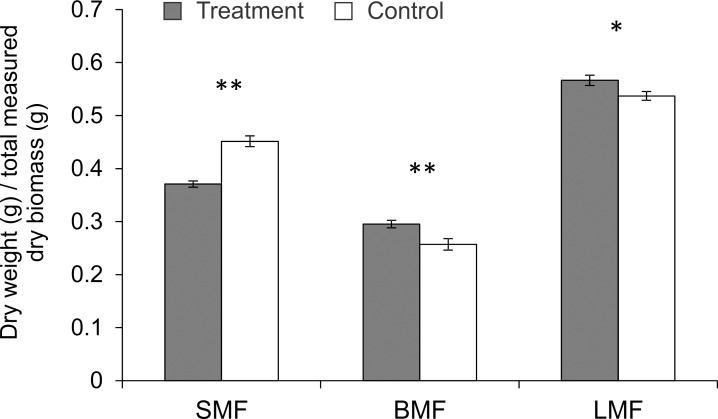
The effect of touching on potato mass fractions (SMF–Stem Mass Fraction, BMF–Branch Mass Fraction and LMF–Leaf Mass Fraction). Significant differences between treatments (*P < 0.05; **P < 0.01; Tukey’s HSD test).

The effects of brief and light touching on potato plants are presented in Table 1. Touching resulted in significant reduction in plant height, stem weight, number of internodes and average branch diameter compared to untouched control plants. Total above ground biomass was not affected by touching, but SMF was reduced, and BMF and LMF significantly increased (Fig 1). Changes in the proportion of biomass of individual organs as a fraction of total above ground biomass suggest a specific pattern of biomass redistribution from the stem to branches and leaves.

**Table 1:** Effect of one minute of touching over a period of 17 days on morphological characteristics of potato plants.

	Mean Touched			Mean Control			p value
Leaf surface (cm^2^)	4498.22	±	165.29	3952.41	±	162.33	**0.02**
Leaf weight (g)	5.91	±	0.22	5.53	±	0.15	0.16
Number of leafs	258.05	±	13.98	248.65	±	10.14	0.59
SLA	767.67	±	24.20	731.61	±	26.01	0.32
Plant height (cm)	22.86	±	0.78	32.41	±	2.31	**<0.01**
Stem weight (g)	1.48	±	0.10	2.13	±	0.12	**<0.01**
Number of internodes	11.20	±	0.58	13.45	±	0.29	**<0.01**
Branch number	32.40	±	1.15	32.45	±	1.57	0.98
Branch weight (g)	3.19	±	0.24	2.70	±	0.18	0.10
Total branch length (cm)	564.02	±	23.56	506.86	±	20.15	0.07
Branch diameter (mm)	4.69	±	0.13	5.36	±	0.13	**<0.01**
Number of tubers/plant	4.45	±	0.71	4.10	±	0.31	0.65
Tuber fresh weight/plant (g)	12.15	±	1.50	12.58	±	1.48	0.84
Total above ground weight (g)	10.59	±	0.54	10.36	±	0.35	0.72

Leaf surface of touched plants was significantly higher than untouched plants (Table 1). The number of tubers and their fresh weight were not affected by touching. As a result of the biomass changes, touched plants were shorter with a more compact appearance.

### Effects of touching on trichomes and pavement cells

**Fig 2:**
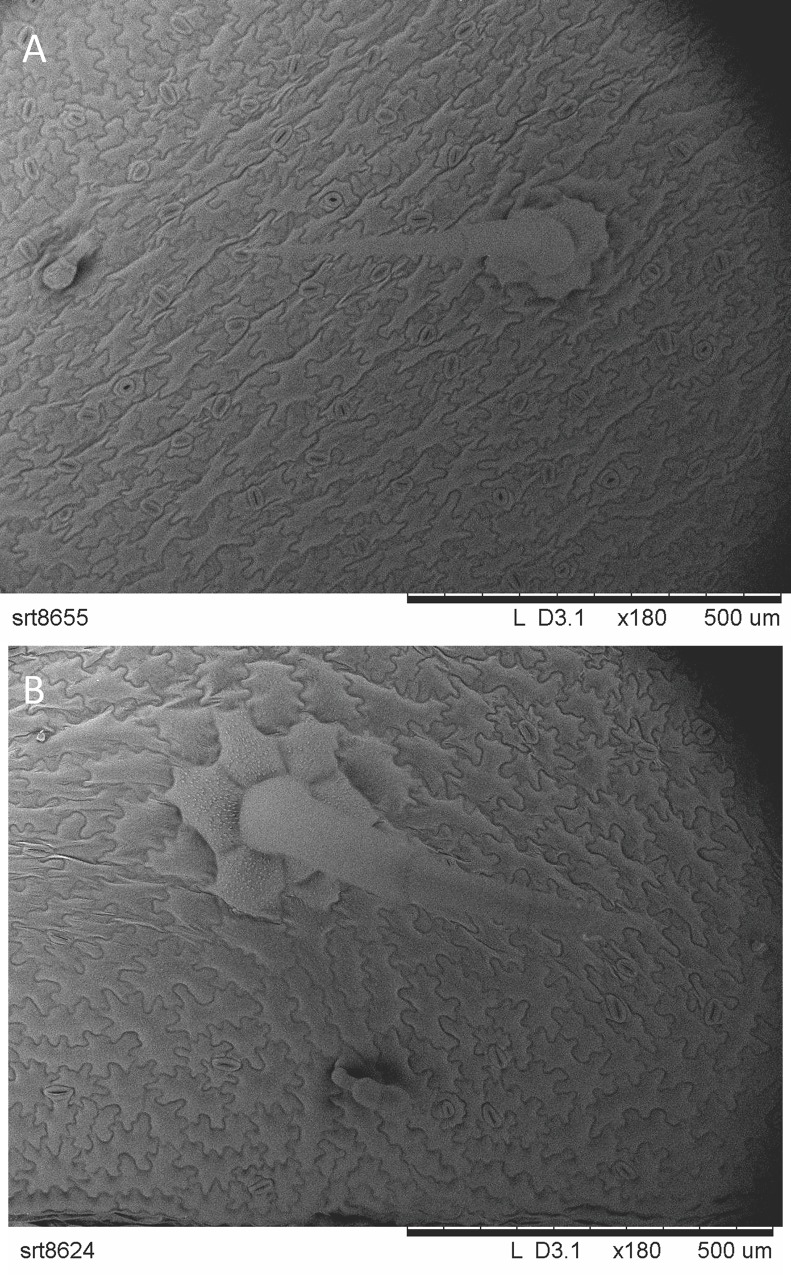
Potato non glandular trichomes obtained by SEM under magnification ×180 on leaf surface from A) touched terminal leaf, B) untouched terminal leaf. No tissue damage was observed on leaf samples.

**Fig 3:**
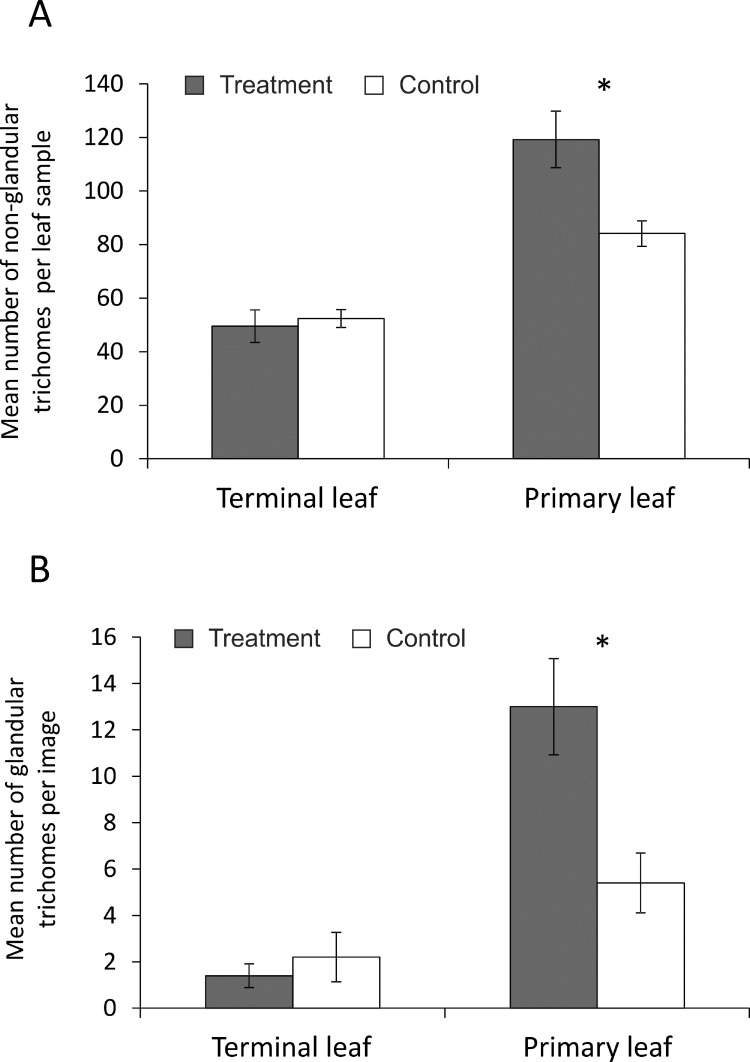
Mean number of: A) non-glandular trichomes per leaf sample on images obtained by light microscopy, B) glandular trichomes per image obtained by SEM. Significant differences in the number of trichomes (*P < 0.05; Tukey’s HSD test).

The light and brief touching did not cause trichome injury on treated terminal leaves (Fig 2A and 2B). Two-way ANOVA analyses did not show significant differences in the number of trichomes between treatments (non-glandular F_1, 36_ = 3.78; P = 0.06; glandular F_1, 16_ = 4.37; P = 0.053). However, the interaction between treatment and the type of leaf was significant (non-glandular F_1, 36_ = 7.17; P = 0.01; glandular F_1, 16_ = 8.08; P = 0.01). Post hoc comparisons with Tukey’s HSD tests showed that primary untreated leaves on branches with touched terminal leaves had significantly higher numbers of both types of trichome than the equivalent leaves of control plants (non-glandular P = 0.012) (Fig 3A) (glandular P = 0.015) (Fig 3B).

**Fig 4:**
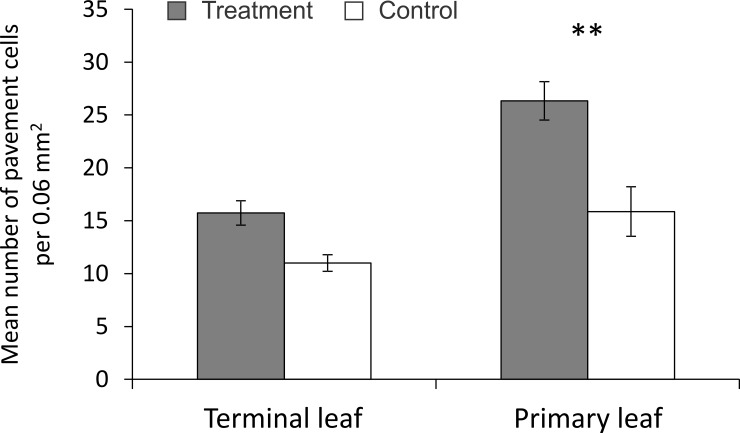
Mean number of pavement cells per 0.06 mm^2^ on image obtained by SEM. Significant differences in the number of trichomes (*P < 0.05; **P < 0.01; Tukey’s HSD test).

One way ANOVA analyses showed significant differences between treatments in the number of pavement cells (F_1, 16_ = 16.12; P = 0.00004). Post hoc comparisons with Tukey’s HSD tests showed that primary untreated leaves on branches with touched terminal leaves had significantly more pavement cells than the equivalent leaves of control plants (P = 0.005) (Fig 4).

### Changes in volatile emission

**Fig 5:**
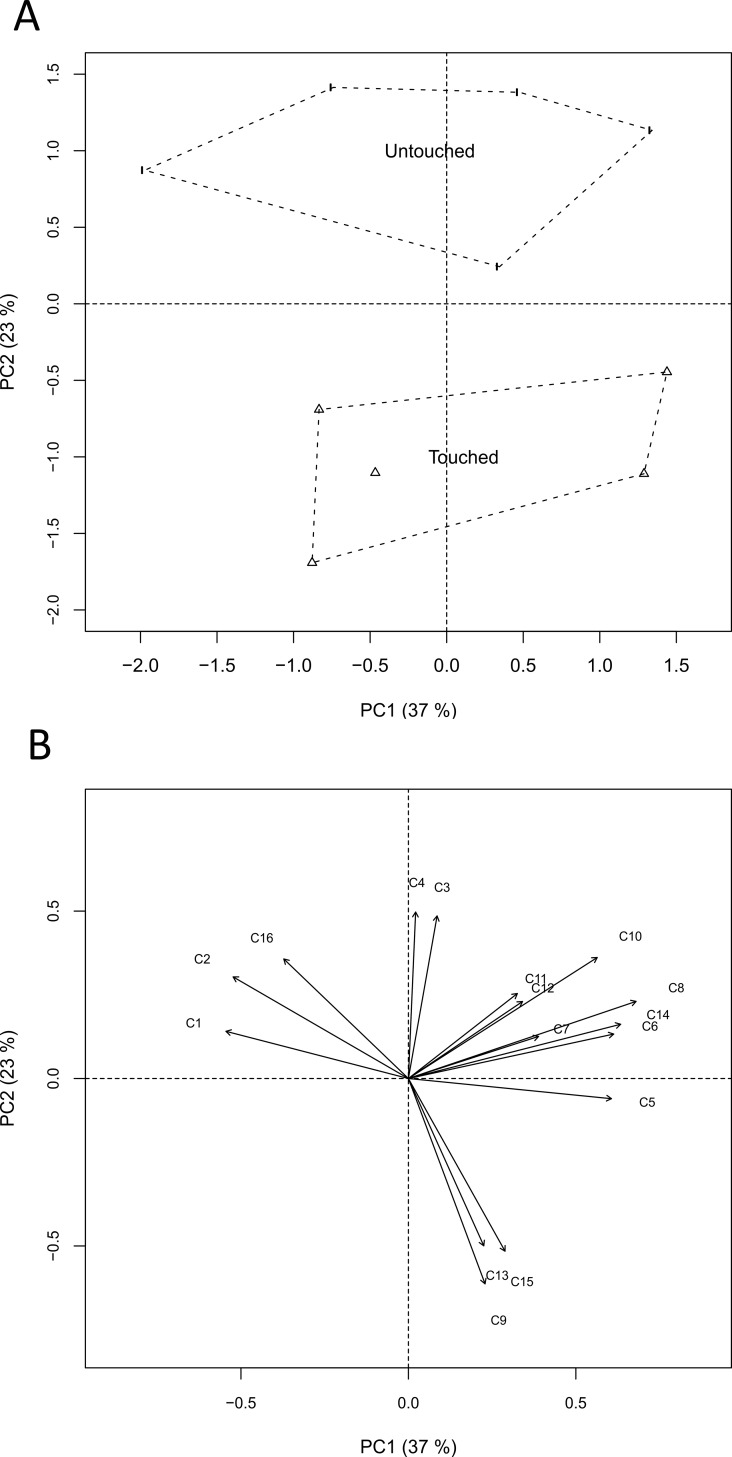
Principal Component analysis (PCA) score plots for touched and control plants. A) Plots PC1 vs PC2 compare total variation in volatile profile between treatments. B) Vectors on the loading plot based on the first two PC show the relative importance of each peak area in volatile compounds released that discriminate treatments (C1: (a)-pinene, C2: 6-methyl-5-hepten-2-one, C3: (E)-ocimene, C4: linalool, C5: DMNT, C6: methyl salicylate, C7: (-)-cyclosativene, C8: a-copaene, C9: (E)-caryophyllene, C10: unknown sesquiterpene 1, C11: unknown sesquiterpene 2, C12: cadinene, C13: (E)-nerolidol, C14: TMTT, C15: germacrene D 4-ol, C16: hexahydrofarnesyl acetone).

Compounds identified in headspace collections from touched and control potato plants are shown in Table 2. PCA revealed variation in the emission of volatile compounds between treatments. PC2 distinguished the treatments better than PC1, and shows clear separation of touched from untouched plants (Fig 5A). In particular, the score plot constructed from the principal components PC1 and PC2 cumulatively captured 60% of the total variation in volatile profiles; PC1 accounted for 37% and PC2 for 23% (Fig 5B). The results of PCA resolved which of 16 volatile compounds were discriminated best among treatments. Longer vectors on the loading plots revealed a greater relative contribution of each peak to the principal components (Fig 5B). Eigenvector weightings of PC1 were predominantly informed by (E)-caryophyllene (C9), (E)-nerolidol (C13) and germacrene D-4-ol (C15). The statistical analyses of individual compounds of headspace of potato previously exposed to touching identified and quantified five individual volatile compounds associated with the touching treatment (Table 2). Significantly higher amounts of (E)-caryophyllene (P = 0.002), (E)-nerolidol (P = 0.008) and germacrene D-4-ol (P = 0.02) were released by touched plants compared to controls, whereas the emission of (E)-ocimene (P = 0.03) and linalool (P = 0.03) significantly decreased. Touched plants showed no significant change in the total amount of volatiles released compared to control plants (P = 0.96).

**Table 2:** Volatile compounds released by treated and control plants (p values from T-test). TMTT: (E,E)-4,8,12-Trimethyl-1,3,7,11-tridecatetraene, DMNT: (E)-4,8-dimethyl-1,3,7-nonatriene.

Compound	Chemical name	Treatment Mean±SE	Control Mean±SE	P value
C1	(a)-pinene	1.55±0.92	2.26±1.33	0.67
C2	6-methyl-5-hepten-2-one	1.69±0.61	3.16±1.17	0.30
C3	(E)-ocimene	1.26±0.28	3.03±0.62	**0.03**
C4	linalool	5.11±0.88	9.32±1.37	**0.03**
C5	DMNT	25.32±10.00	22.21±6.63	0.80
C6	methyl salicylate	9.45±3.00	10.65±4.20	0.82
C7	(-)-cyclosativene	1.19±0.34	1.13±0.20	0.87
C8	α-copaene	26.82±6.33	30.79±7.01	0.69
C9	(E)-caryophyllene	16.60±2.93	2.84±0.73	**0.002**
C10	unknown sesquiterpene 1	1.56±0.41	2.05±0.47	0.45
C11	unknown sesquiterpene 2	5.89±1.02	6.89±1.31	0.57
C12	cadinene	2.64±0.43	3.25±0.79	0.52
C13	(E)-nerolidol	1.38±0.18	0.55±0.15	**0.008**
C14	TMTT	38.26±11.29	45.80±10.86	0.64
C15	germacrene D 4-ol	10.00±3.42	0.49±0.23	**0.02**
C16	hexahydrofarnesyl acetone	7.85±0.77	10.17±1.91	0.29

### Aphid olfactory response

**Fig 6:**
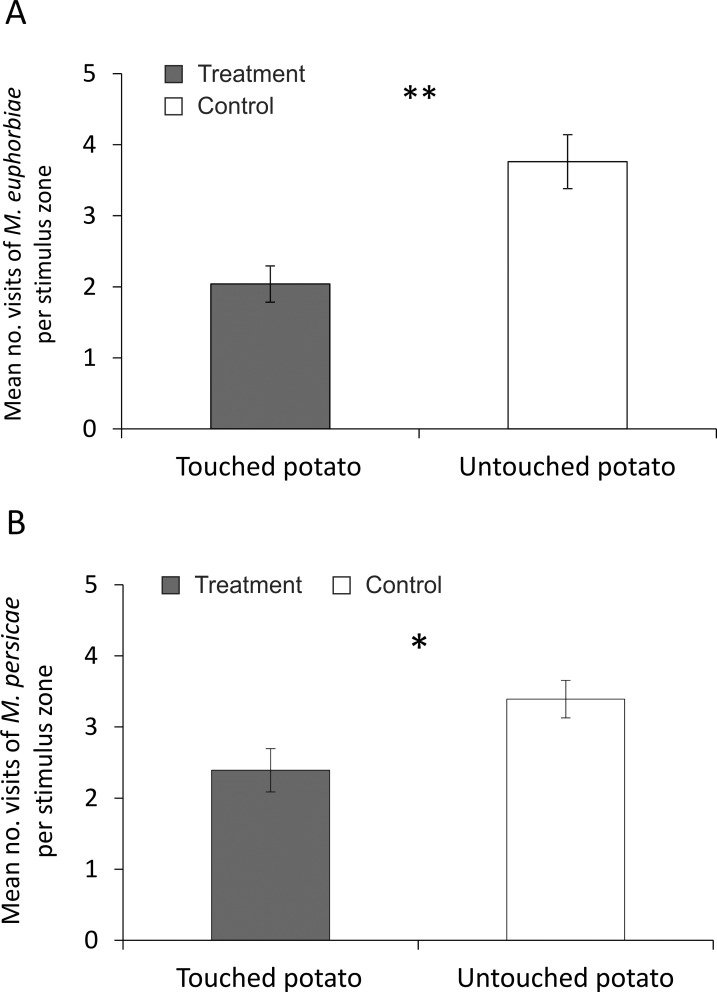
Olfactory preferences of: A) *M*. *euphorbiae* and B) *M*. *persicae* for volatiles released from touched and untouched potato plants. Significant differences in aphid olfactory preference (*P < 0.05, **P < 0.01; Wilcoxon’s mean pairs test).

Both aphid species showed a significantly lower preference (measured as mean number of visits in odour field) for odour of touched potato plants compared to untreated plants: *M*. *euphorbiae* (Wilcoxon’s test, N = 22; Z = 2.78; P = 0.005) (Fig 6A) and *M*. *persicae* (Wilcoxon’s test, N = 18; Z = 2.26; P = 0.02) (Fig 6B).

## Discussion

Our results show that one minute of light touching of potato leaves, can induce changes in biomass allocation and increase trichome production on adjacent leaves via a systemic effect. The touching also induced changes in the blend of volatile organic compounds released by plants, and this resulted in reduced olfactory attraction of insect herbivores. The results suggest that plant responses to even light contact can reprogram the growth of individual organs.

### Plant adaption to touch stimuli

The ability of plants to modify their growth and morphology is fundamental to reproductive performance and fitness [37]. A recent analysis of plant biomass distribution in 1200 species found that *Solanaceae* have a well-developed capacity to distribute biomass among above ground parts [38]. Our results show that application of light mechanical stimuli simulating touching between leaves of neighbouring plants induces changes in the growth pattern of potato. Touching modified the pattern of biomass distribution, reducing SMF and increasing BMF and LMF. This is a common plant response to mechanical stimulation observed in other species [14, 39, 40]. The brief touching of terminal potato leafs also affected above ground biomass distribution. As a result, the number of internodes was reduced, making touched plants more compact with increased radial expansion. Further, compact plants are more resistant to mechanical stress and potential mechanical damage caused by environmental factors such as wind [41, 42], but the response may also represent an adaptation to meet competition for space from neighbouring plants. It has been shown that touching reduces leaf surface area [43] which may depress plant competitiveness with neighbours [21, 44]. In contrast, the increase in leaf surface on touched plants observed in our study suggests that brief and light contact with neighbours can potentially induce responses that prepare plants for imminent competition.

The changes in trichome number observed on touched plants did not affect the number and average weight of fresh tubers per plant. These findings are consistent with those of Kaplan *et al*. who found no evidence for trade-offs between potato tuber yield and higher trichome production [45]. In contrast, a positive correlation between trichome density and soybean yield has been demonstrated [46]. Plant stress induced by damage or drought can increase overall trichome density [47]. In our study an increase was not observed on the touched, terminal leaves, but rather on untouched primary leaves on the same branches as the touched leaves. It has been shown that trichomes, one of the most fragile structures on the leaf surface, can be damaged by gentle forms of mechanical stimulation [48]. However, we did not find evidence that our touching treatment damaged trichomes. One minute per day of light touching was enough to increase the production of glandular trichomes on untouched leaves of treated plants. A similar systemic enhancement of trichome production on undamaged leaves has been observed after insect feeding [49, 50], suggesting that it may be a common plant response to stress. An expected reduction in trichome occurrence due to a dilution effect caused by increase in the leaf surface of touched plants was not observed.

Trichomes on the leaf surface may play multiple functional roles important for plant growth. For example, increase in trichome production is negatively correlated with transpiration rate [51, 52], implying that the presence of trichomes can be important in reducing water loss. Trichomes can also reduce the absorption of sunlight, dissipate absorbed heat and decrease the rate of carbon dioxide diffusion [53], thereby interfering with the rate of photosynthesis [54, 55]. Thus, increase in the number of trichomes suggests that light, periodical contact between plants may have implications for plant resource use. Greater leaf reflectivity can reduce transpiration, because it lessens the solar radiation load on the sunlit canopy surface, and can also enhance photosynthesis if more radiation is reflected to the shaded leaves in the lower part of the canopy. In light of these leaf morphological changes, touched plants are more likely to outperform untouched neighbours in resource poor habitats. It is generally considered that plants adapted to such habitats are expected to deploy conservative resource-use strategies (characterized by more efficient resource use), but at the expense of fast growth [56].

### Insect response to touch stimulated plants

The brief and light touching resulted in significant changes in emission of volatile organic compounds by the plant. A similar effect has been found in maize and bean plants [28]. Three sesquiterpene compounds were released in higher amounts by touched plants, (E)-caryophyllene, (E)-nerolidol and germacrene-4D-ol, and release of two terpenes (E)-ocimene and linalool was reduced. Most of the compounds identified in our study have been previously detected from potato leaves [34, 35]. Potato trichomes are known to contain sesquiterpenes, including (E)-caryophyllene [57], and the increase in these compounds could be related directly to the touching of terminal leaves and/or to the systemic increase in trichomes on untouched primary leaves. The decrease in emission of the two terpenes is less likely to be due directly to touching itself, and could be related to changes in the plant’s underlying physiology.

Plant volatiles are used by arthropod herbivores to locate hosts, discriminate hosts from non-hosts and gain information on plant quality and condition [58]. For aphids volatiles play a vital role in the host location process [59, 60]. In our study, *M*. *euphorbiae* and *M*. *persicae* were less attracted to odour of touched plants than to odour of untouched plants. This could be due to aphid responses to individual compounds such as (E)-nerolidol, which has been shown to repel *M*. *persicae* [5]. It has been also shown that increased levels of (E)-nerolidol and (E)-β-caryophyllene within volatile blends can repel herbivores and enhance the recruitment of biological control agents [61–63]. Since aphids are known to discriminate small changes in ratios of volatile compounds [60], the changes in the overall blend induced by touching in our study may be responsible for the effects on aphid attraction. Thus plant mechanical interaction may have ecological effects beyond the plant itself, affecting organisms at higher trophic levels. This may be a parallel to plant-plant interaction via chemicals, which has been shown to have extensive effects on insects [25, 64].

Leaf trichomes may also serve as mechanoreceptors or sensors of insect movement on the leaf surface even in the absence of leaf rupture. Insect contact with trichomes on the leaf was shown to be sufficient to up-regulate defence transcripts and activate defences that provide extra protection against newly hatched larvae [65, 66]. Such an early detection system of light mechanical stimuli may induce plant defences against subsequent herbivory or other herbivore species that later colonize the host [65, 67]. An increase in trichome frequency can create a mechanical barrier that interferes with insect movement and redirects damage from the most valuable parts of the plant to less valuable parts [55], or to nearby plants. On the other hand, trichomes present an important factor that provides resistance to viruses by reducing the maximum incidence of virus infection [68].

The number of pavement cells was also increased by touching in our study, showing that the epidermal cells structure that provides mechanical strength and protection for inner tissues was also affected by the treatment. The pavement cells are a barrier for most piecing insects that puncture the leaves between epidermal cells such as aphids [69].

## Concluding remarks

Interaction between plants by light touching can regulate patterns of biomass distribution between different organs, enabling plants to establish a competitive above ground habitus. The capacity of potato plants to respond to touching may potentially be a trait that enables them to redirect growth into organs from which they may benefit most in competitive situation with neighbours. These responses appear to induce the side effects of making potato plants less attractive to aphids through the changes in released volatile blend. Our findings, together with other recent studies [15, 28], suggest a potential for broader implications of plant responses to mechanical stimuli, extending to organisms at higher trophic levels. The ecological and evolutionary significance of these effects in nature remain to be investigated.

## Supporting Information

S1 Fig Terminal leaves marked by circles were exposed to brief mechanical stimuli induced by a soft face brush. (TIF)

S2 Fig Graphical illustration of potato branch from a treated plant showing the leaves from which samples were taken. (TIF)

S1 Table Morphological indices of touched and untouched potato. (XLSX)

S2 Table Biomass fractions of touched and untouched potato. (XLSX)

S3 Table The number of glandular and non-glandular trichomes on touched and untouched potato leaves. (XLSX)

S4 Table Mean number of pavement cells on touched and untouched potato leaves. (XLSX)

S5 Table Volatiles released by touched and untouched potato. (XLSX)

S6 Table Olfactory response of *M*. *euphorbiae* to volatiles released by touched and untouched potato. (XLSX)

S7 Table Olfactory response of *M*. *persicae* to volatiles released by touched and untouched potato. (XLSX)

## References

[1] Franklin KA. Shade avoidance. New Phytol. 2008; 179: 930–944. 10.1111/j.1469-8137.2008.02507.x 18537892

[2] Izaguirre MM, Mazza CA, Biondini M, Baldwin IT, Ballaré CL. Remote sensing of future competitors: impacts on plant defenses. PNAS 2006; 103: 7170–7174. 10.1073/pnas.0509805103 16632610 PMC1459035

[3] Gagliano M, Mancuso S, Robert D. Towards understanding plant bioacoustics. Trends Plant Sci. 2012; 17: 323–325. 10.1016/j.tplants.2012.03.002 22445066

[4] Biedrzycki ML, Jilany TA, Dudley SA, Bais HP. Root exudates mediate kin recognition in plants. Commun. Integr. Biol. 2010; 3: 1–8. 20539778 10.4161/cib.3.1.10118 PMC2881236

[5] Ninkovic V, Dahlin I, Vucetic A, Petrovic-Obradovic O, Glinwood R, Webster B. Volatile exchange between undamaged plants—a new mechanism affecting insect orientation in intercropping. PLoS ONE. 2013; 8(7): e69431 10.1371/journal.pone.0069431 23922710 PMC3726678

[6] Salter MG, Franklin KA, Whitelam GC. Gating of the rapid shade-avoidance response by the circadian clock in plants. Nature 2003; 426: 680–683. 10.1038/nature02174 14668869

[7] Ninkovic V. Volatile communication between barley plants affects biomass allocation. J. Exp. Bot. 2003; 54: 1931–1939. 10.1093/jxb/erg192 12815028

[8] Choh Y, Shimoda T, Ozawa R, Dicke M, Takabayashi J. Exposure of lima bean leaves to volatiles from herbivore-induced conspecific plants results in emission of carnivore attractants: active or passive process? J. Chem. Ecol. 2004; 30: 1305–1317. 15503521 10.1023/b:joec.0000037741.13402.19

[9] Kegge W, Ninkovic V, Glinwood R, Welschen RAM, Voesenek LACJ, Pierik R. Red:far-red light conditions affect the emission of volatile organic compounds from barley (*Hordeum vulgare*), leading to altered biomass allocation in neighbouring plants. Ann. Bot. 2015; 115: 961–970. 10.1093/aob/mcv036 25851141 PMC4407068

[10] Moulia B, Coutand C, Lenne C. Posture control and skeletal mechanical acclimation in terrestrial plants: implication for mechanical modelling of plant architecture. Am. J. Bot. 2006; 93: 1477–1489. 10.3732/ajb.93.10.1477 21642095

[11] Telewski FW. A unified hypothesis of mechanopreception in plants. Am. J. Bot. 2006; 93: 1306–1316. 21642094 10.3732/ajb.93.10.1466

[12] Braam J. In touch: plant responses to mechanical stimuli. New Phytol. 2005; 165: 373–389. 10.1111/j.1469-8137.2004.01263.x 15720650

[13] Forterre Y, Skothelm JM, Dumals J, Mahadevan L. How the Venus flytrap snaps. Nature 2005; 433: 421–425. 10.1038/nature03185 15674293

[14] Liu Y, Schieving F, Stuefer JF, Anten NPR. The effects of mechanical stress and spectral shading on the growth and allocation of ten genotypes of a stoloniferous plant. Ann. Bot. 2007; 99: 121–130. 10.1093/aob/mcl230 17085473 PMC2802973

[15] Chehab W, Yao C, Henderson Z, Kim Se, Braam J. Arabidopsis touch-induced morphogenesis is jasmonate mediated and protects against pests. Curr. Biol. 2012; 22: 701–706. 10.1016/j.cub.2012.02.061 22483939

[16] Anten NPR, Casado-Garcia R, Nagashima H. Effects of mechanical stress and plant density on mechanical characteristics, growth, and lifetime reproduction of tobacco plants. Am. Nat. 2005; 166: 650–660. 10.1086/497442 16475082

[17] Saidi I, Ammar S, Demont-Caulet N, Thévenin J, Lapierre C, Bouzid S et al Thigmomorphogenesis in *Solanum lycopersicum*. Plant Signal. Behav. 2010; 5: 122–125. 20009518 10.4161/psb.5.2.10302 PMC2884111

[18] Braam J, Davis RW. Rain-, wind-, and touch-induced expression of calmodulin and calmodulin-related genes in Arabidopsis. Cell 1990; 60: 357–364. 2302732 10.1016/0092-8674(90)90587-5

[19] de Wit M, Kegge W, Evers JB, Vergeer-van Eijka MH, Gankema P, Voeseneka LACJ et al Plant neighbor detection through touching leaf tips precedes phytochrome signals. PNAS 2012; 109: 14705–14710. 10.1073/pnas.1205437109 22908260 PMC3437826

[20] Stolarz M. Circumnutation as a visible plant action and reaction. Plant Signal. Behav. 2009; 4: 380–387. 10.4161/psb.4.5.8293 19816110 PMC2676747

[21] Whippo CW. Phototropism: Bending towards Enlightenment. Plant Cell 2006; 18: 1110–1119. 10.1105/tpc.105.039669 16670442 PMC1456868

[22] Pettersson J, Tjallingii WF, Hardie J. Host-plant selection and feeding In: van Emden H, Harrington R, editors. Aphids as crop pest, vol 4 CAB International, Wallingford; 2007 pp. 87–113.

[23] Moran PJ, Cipollini DF Jr. Effect of wind-induced mechanical stress on soluble peroxidase activity and resistance to pests in cucumber. J. Phytopathol. 1999; 147: 313–316.

[24] Dahlin I, Ninkovic V. Aphid performance and population development on their host plants is affected by weed–crop interactions. J. Appl. Ecol. 2013; 50: 1281–1288.

[25] Karban R, Yang LH, Edwards KF. Volatile communication between plants that affects herbivory: a meta-analysis. Ecol. Lett. 2014; 17: 44–52. 10.1111/ele.12205 24165497

[26] DeFauw SL, He Z, Larkin RP, Mansour SA. Sustainable Potato Production and Global Food Security In: He Z, Larkin R, Honeycutt W, editors. Sustainable potato production. Global case studies. Springer, Dordrecht, Heidelberg, New York, London, 2012 pp. 3–19.

[27] Jeffries C, Barker H, Khurana SMP. Viruses and viroids In: Goplan J, Khurana SMP, editors. Handbook of potato production, improvement, and postharvest management. Food Products Press, New York, USA; 2006 pp. 387–448.

[28] Markovic D, Glinwood R, Olsson U, Ninkovic V. Plant response to touch affects the behaviour of aphids and ladybirds. Arthropod Plant Interact. 2014; 8: 171–181.

[29] Montgomery JA, Bressan RA, Mitchell CA. Optimizing environmental conditions for mass application of mechano-dwarfing stimuli to Arabidopsis. J. Am. Soc. Hortic. Sci. 2004; 129: 339–343. 15776543

[30] Anten NPR, Alcala-Herrera R, Schieving F, Onoda Y. Wind and mechanical stimuli differentially affect leaf traits in *Plantago major*. New Phytol. 2010; 188: 554–564. 10.1111/j.1469-8137.2010.03379.x 20663062

[31] Pieczynski M, Marczewski W, Hennig J, Dolata J, Bielewicz D, Piontek P et al Down-regulation of CBP80 gene expression as a strategy to engineer a drought-tolerant potato. Plant Biotechnol. J. 2013; 11: 459–469. 10.1111/pbi.12032 23231480

[32] CorelDRAW X5 –Corel Corporation, Release 2010.

[33] Glas JJ, Schimmel BCJ, Alba JM, Escobar-Bravo R, Schuurink RC, Kant MR. Plant Glandular Trichomes as Targets for Breeding or Engineering of Resistance to Herbivores. Int. J. Mol. Sci. 2012; 13: 17077–17103. 10.3390/ijms131217077 23235331 PMC3546740

[34] Weissbecker B, van Loon JJA, Posthumus MA, Bouwmeester HJ, Dicke M. Identification of volatile potato sesquiterpenoids and their olfactory detection by the two-spotted stinkbug *Perillus bioculatus*. J. Chem. Ecol. 2000; 26: 1433–1445.

[35] Szafranek B, Chrapkowska K, Pawińska M, Szafranek J. Analysis of leaf surface sesquiterpenes in potato varieties. J. Agric. Food Chem. 2005; 53: 2817–2822. 10.1021/jf040437g 15826024

[36] Dell Inc. 2015. Dell Statistica, version 13.dell.com.

[37] Read J, Stokest A. Plant biomechanics in an ecological context. Am. J. Bot. 2006; 93: 1546–1565. 10.3732/ajb.93.10.1546 21642101

[38] Poorter H, Jagodzinski AM, Ruiz-Peinado R, Kuyah S, Luo Y, Oleksyn Jet al. How does biomass distribution change with size and differ among species? An analysis for 1200 plant species from five continents. New Phytol. 2015; 208: 736–749. 10.1111/nph.13571 26197869 PMC5034769

[39] Niklas KJ. Effect of vibration on mechanical properties and biomass allocation pattern of *Capsella bursa-pastoris* (Cruciferae). Ann. Bot. 1998; 82: 147–156.

[40] Pigliucci M. Touchy and bushy: phenotypic plasticity and integration in response to wind stimulation in *Arabidopsis thaliana*. Int. J. Plant. Sci. 2002; 163: 399–408.

[41] Coutand C, Dupraz C, Jaouen G, Ploquin S, Adam B. Mechanical stimuli regulate the allocation of biomass in trees: demonstration with young *Prunus avium* trees. Ann. Bot. 2008; 101: 1421–1432. 10.1093/aob/mcn054 18448448 PMC2710262

[42] Jaffe MJ, Leopold AC, Staples RC. Thigmo responses in plants and fungi. Am. J. Bot. 2002; 89: 375–382. 10.3732/ajb.89.3.375 21665632

[43] Zhang Z, Zhang X, Wang S, Xin W, Tang J, Wang Q. Effect of Mechanical Stress on Cotton Growth and Development. PLoS ONE 2013; 8(12): e82256 10.1371/journal.pone.0082256 24363813 PMC3868487

[44] Falster DS, Westoby M. Plant height and evolutionary games. Trends Ecol. Evol. 2003; 18: 337–343.

[45] Kaplan I, Dively GP, Denno RF. The costs of anti-herbivore defense traits in agricultural crop plants: a case study involving leafhoppers and trichomes. Ecol. Appl. 2009; 19 (4): 864–872. 19544730 10.1890/07-1566.1

[46] Zhang J, Specht JE, Graef GL, Johnson BE. Pubescence density effects on soybean seed yield and other agronomic traits. Crop Sci. 1992; 32: 641–646.

[47] Gonzáles WL, Negritto MA, Suárez LH, Gianoli E. Induction of glandular and non-glandular trichomes by damage in leaves of *Madia sativa* under contrasting water regimes. Acta Oecol. 2008; 33: 128–132.

[48] Benikhlef L, L’Haridon F, Abou-Mansour E, Serrano M, Binda M, Costa A et al Perception of soft mechanical stress in Arabidopsis leaves activates disease resistance. BMC Plant Biol. 2013; 13:133–145. 10.1186/1471-2229-13-133 24033927 PMC3848705

[49] Traw MB, Dawson TE. Reduced performance of two specialist herbivores (Lepidoptera: Pieridae, Coleoptera: Chrysomelidae) on new leaves of damaged black mustard plants. Environ. Entomol. 2002; 31: 714–722.

[50] Holeski LM. Within and between generation phenotypic plasticity in trichome density of *Mimulus guttatus*. J. Evol. Biol. 2007; 20: 2092–2100. 10.1111/j.1420-9101.2007.01434.x 17903186

[51] Pérez-Estrada LB, Cano-Santana Z. Oyama K. Variation in leaf trichomes of *Wigandia urens*: environmental factors and physiological consequences. Tree Physiol. 2000; 20: 629–632. 12651428 10.1093/treephys/20.9.629

[52] Benz BW, Martin CE. Foliartrichomes, boundarylayers, and gas exchange in 12 species of epiphytic Tillandsia (Bromeliaceae). J. Plant. Physiol. 2006; 163: 648–656. 10.1016/j.jplph.2005.05.008 16545998

[53] Galmés J, Medrano H, Flexas J. Photosynthesis and photo inhibition in response to drought in a pubescent (var. *minor*) andaglabrous (var. *palaui*) variety of *Digitalis minor*. Environ. Exp. Bot. 2007; 60: 105–111.

[54] Eller BM, Willi P. The significance of leaf pubescence for the absorption of global radiation by *Tussilago farfara* L. Oecologia 1977; 29: 179–187. 28308649 10.1007/BF00345796

[55] Woodman RL, Fernandes GW. Differential mechanical defense: herbivory, evapotranspiration, and leaf-hairs. Oikos 1991; 60: 11–19.

[56] Aerts R, Chapin FS. The mineral nutrition of wild plants revisited: a re-evaluation of processes and patterns. Adv. Ecol. Res. 1999; 30: 1–67.

[57] Avé DA, Gregory P, Tingey WM. Aphid repellent sesquiterpenes in glandular trichomes of *Solanum berthaultii* and *S*. *tuberosum*. Entomol. Exp. Appl. 1987; 44: 131–138.

[58] Baldwin IT. Plant volatiles. Curr. Biol. 2010; 20(9): R392–397. 10.1016/j.cub.2010.02.052 20462477

[59] Pickett JA, Glinwood R. Chemical Ecology In: van Emden H, Harrington R, editors. Aphids as crop pests. CAB International, Wallingford; 2007 pp. 235–254.

[60] Webster B. The role of olfaction in aphid host location. Physiol. Entomol. 2012; 37: 10–18.

[61] Cook S, Khan ZR, Pickett JA. The use of push-pull strategies in Integrated Pest Management. Annu. Rev. Entomol. 2007; 52: 375–400. 10.1146/annurev.ento.52.110405.091407 16968206

[62] Köllner TG, Held M, Lenk C, Hiltpold I, Turlings TCJ, Gershenzon J et al Maize (E)-β-Caryophyllene synthase implicated in indirect defense responses against herbivores is not expressed in most American maize varieties. Plant Cell 2008; 20: 482–494. 10.1105/tpc.107.051672 18296628 PMC2276456

[63] Kos M, Houshyani B, Overeem AJ, Bouwmeester HJ, Weldegergis BT, van Loon JJA et al Genetic engineering of plant volatile terpenoids: effects on a herbivore, a predator and a parasitoid. Pest Manag. Sci. 2013; 69: 302–311. 10.1002/ps.3391 22933413

[64] Glinwood R, Ninkovic V, Pettersson J. Chemical interaction between undamaged plants–Effects on herbivores and natural enemies. Phytochemistry 2011; 72: 1683–1689. 10.1016/j.phytochem.2011.02.010 21388645

[65] Peiffer M, Tooker JF, Luthe DS, Felton GW. Plants on early alert: glandular trichomes as sensors for insect herbivores. New Phytol. 2009; 184(3): 644–656. 10.1111/j.1469-8137.2009.03002.x 19703113

[66] Sato Y, Kawagoe T, Sawada Y, Hirai MY, Kudoh H. Frequency -dependent herbivory by a leaf beetle, *Phaedon brassicae*, on hairy and glabrous plants of *Arabidopsis halleri* subsp. *gemmifera*. Evol.Ecol. 2014; 28: 545–559.

[67] Valkama E, Koricheva J, Salminen JP, Helander M, Saloniemi I, Saikkonen Ket al. Leaf surface traits: overlooked determinants of birch resistance to herbivores and foliar micro-fungi? Trees (Berlin) 2005; 19: 191–197.

[68] Ren Q, Pfeiffer TW, Ghabrial SA. Relationship between soybean pubescence density and soybean mosaic virus field spread. Euphytica 2000; 111(3): 191–198.

[69] Farmer E. Leaf defence Oxford, UK: Oxford University Press, 2014 pp. 48.

