# Supplementary figures and images for: Plant Responses to Brief Touching: A Mechanism for Early Neighbour Detection?
Source: PLoS One. 2016 Nov 9;11(11):e0165742. doi: 10.1371/journal.pone.0165742 (PMC5102373; doi:10.1371/journal.pone.0165742)

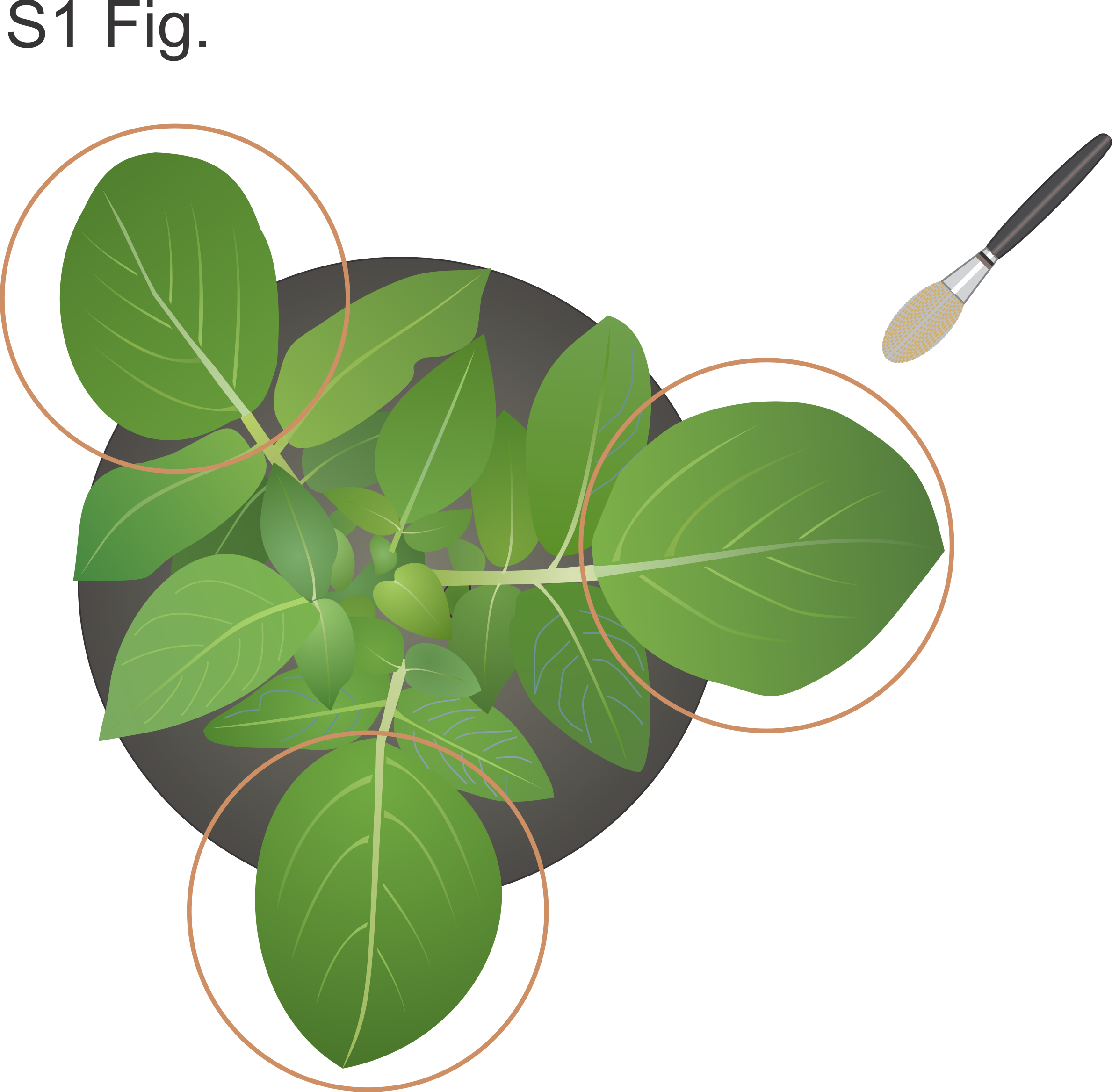

Supplement: S1 Fig — (TIF) [file pone.0165742.s001.tif]

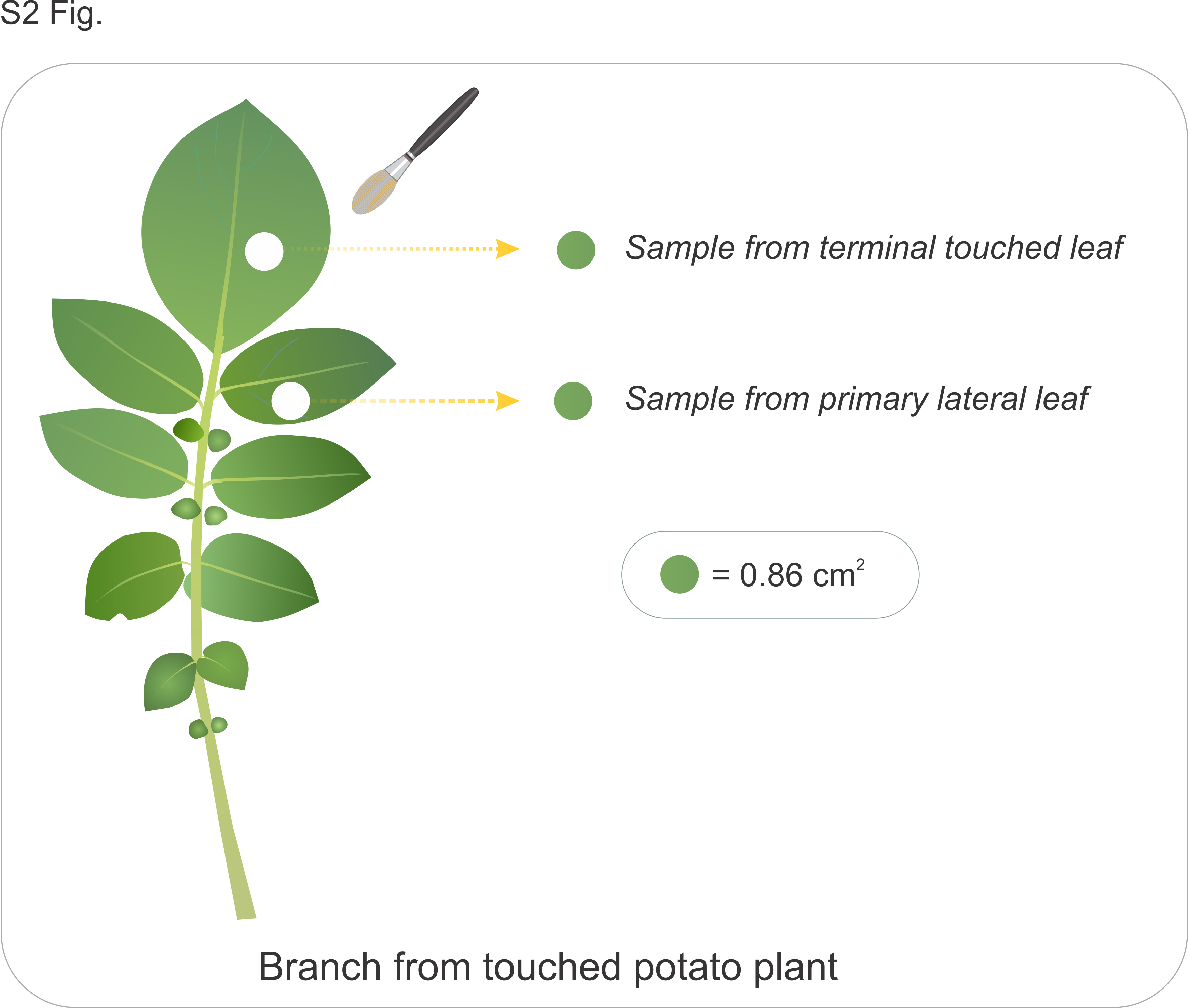

Supplement: S2 Fig — (TIF) [file pone.0165742.s002.tif]
